# The correlation between sadomasochists' experience and their sadomasochistic behaviors and fantasies: A qualitative analysis of interviews

**DOI:** 10.1002/pchj.706

**Published:** 2023-12-17

**Authors:** Wanying Liang, Yuqing Zhang

**Affiliations:** ^1^ Key Laboratory of Mental Health Institute of Psychology, Chinese Academy of Sciences Beijing China; ^2^ Department of Psychology University of Chinese Academy of Sciences Beijing China

**Keywords:** behaviors and fantasies, experience, rational model, sadomasochism

## Abstract

Lacking a comprehensive understanding of sadomasochism makes difficulties in judicial dispositions, clinical interventions, and mental health services. This study explores the correlation between sadomasochists' growth experience and their sadomasochistic behaviors and fantasies. We interviewed 51 sadomasochists from a Chinese subcultural website, coded and analyzed the interview records, conducted correlation and cluster analyses on the reference points of the nodes of impressive experience and sadomasochistic behaviors and fantasies, and constructed the model of Experience—Behaviors and Fantasies. We found that sadomasochists' typical impressive experiences are family parenting and sexual experience; sadomasochistic behaviors and fantasies can be classified into five categories: spirit, punishment, sex, canine, and excretion; and sadomasochistic behaviors and fantasies are partially correlated with sadomasochists' impressive experiences, indicating psychoanalytic theory is the leading theory for the driving processes of sadomasochism, while behaviorist and Gestalt theories also contribute.

## INTRODUCTION

Sadomasochism, or BDSM (which represents three overlapping acronyms: BD [bondage and discipline], DS [domination and submission], and SM [sadomasochism]), has received increasing attention in society and academia. These terms refer to a variety of fantasies and behaviors in that individuals gain sexual arousal and satisfaction by suffering or causing others to suffer, which may be understood as psychological disorder, jurisprudential criminal behavior, sexual orientation, leisure, or sociological subculture (Nitschke, Osterheider, & Mokros, 2009; Nitschke, Ottermann, & Mokro, 2009; Richters et al., 2008; Sprott, 2020; Sprott & Williams, 2019; Stupperich & Strack, 2016). In 1886, psychiatrist Richard von Krafft‐Ebing's introduction of the term “sadomasochism” into psychology laid the groundwork for its umbrella usage to cover consensual sadomasochistic activities alongside nonconsensual sadistic crime and various forms of self‐injury. Since the sexual revolution, practitioners have adopted “SM” to avoid the pathologizing imprint of “sadomasochism.” This is when consensual sadomasochism is distinguished from general sadomasochism. Over the past two decades, “SM” has extended to “SM,” “BDSM,” or “kink” because many communities of people interested in consensual sadomasochism have grown increasingly pansexual, and SM identifications appear to be more fluid than in earlier communities (Newmahr, 2015; Weiss, 2015). Today consensual sadomasochistic play is most often referred to as “BDSM,” or “kink,” which represents non‐pathological sadomasochism, and the practitioners are called “kinksters.” Nonconsensual sadomasochism is still called “sadomasochism,” which tends to refer to pathological sadomasochism, and the practitioners are called “paraphilic” or “sex offenders.” This study uses the term “sadomasochism” to refer to general sadomasochism, covering the pathological and non‐pathological scopes, and the practitioners are called “sadomasochists.” Depending on diagnosis criteria, such as the *Diagnostic and Statistical Manual of Mental Disorders* (5th ed.; *DSM‐5*; American Psychiatric Association, 2014), people can roughly distinguish between pathological and non‐pathological sadomasochism. However, previous research has pointed out that the symptoms of the two are basically similar, though the severity varies (Liang & Zhang, 2020), and found that consensual BDSM communities consist of both pathological and non‐pathological sadists (Erickson & Sagarin, 2021), implying that there is no specific boundary between the two and that studying pathological or non‐pathological sadomasochists brings enlightenment to the other.

### Problems in practice

Previous studies have reported a considerable prevalence of sadomasochistic interests, although the results have been divergent. In a nationally representative study of Belgium, 68.8% of participants reported at least one BDSM fantasy or practice (Holvoet et al., 2017). In a Finnish population‐based adult sample, 38% answered having been interested in BDSM sex (Paarnio et al., 2022). Another nationally representative study from Australia found that only 1.8% of sexually active people (2.2% of men, 1.3% of women) had been involved in BDSM in the previous year (Richters et al., 2008); still, the number is considerable.

Currently, questions remain regarding mental health services, medical care, and judicial work for the sadomasochistic population. Several studies found that sadomasochists do not have more mental health problems than do the general population (Botta et al., 2019; Pascoal et al., 2015; Richters et al., 2008; Wismeijer & Assen, 2013). However, other studies reported that sadomasochists suffer from severe psychological problems (Brown et al., 2017; Roush et al., 2016), indicating that they require psychological counseling or therapy. With the development of online psychological counseling, counselors and therapists are increasingly serving sadomasochists. Unfortunately, sadomasochists have long been discriminated against by the general public and professionals, and the treatment effect for them has been unsatisfactory (Hansen‐Brown & Jefferson, 2022; Kolmes et al., 2006; Lantto & Lundberg, 2021; Sprott et al., 2017). Kelsey et al. (2013) found that about 25% to 30% of sadomasochists are not willing to disclose their sexual interests when seeking professional help because they worry about being resented by the therapists. Moreover, 76% of the therapists were found to have treated at least one client engaged in BDSM; however, only 48% perceived themselves as competent. Therapists should master a comprehensive understanding of the driving process of sadomasochism in order to eliminate their unreasonable discrimination against sadomasochists and provide professional help, while now the situation is the opposite.

Practical judicial work currently lacks a reliable diagnosis to sentence sexual sadists correctly and a targeted treatment for prisoners to reduce recidivism. Current diagnosis of sadomasochism, such as the *DSM*, has been challenged for being too vague (Marshall et al., 2016). A study found that two‐thirds of sadistic sexual offenders had not been identified as sadists before the trial and there were no targeted treatment and rehabilitation programs for them in the high‐security forensic hospital where sexual offenders stay (Nitschke, Osterheider, & Mokros, 2009; Nitschke, Ottermann, & Mokro, 2009). Meanwhile, existing studies have not yet constructed rating scales to evaluate sadism comprehensively and accurately (Marshall et al., 2016), nor have they focused on scales for masochism. Researchers have tried to evaluate existing rating scales for sadists, but they tended to concentrate on sexual offenders (Nitschke, Osterheider, & Mokros, 2009; Nitschke, Ottermann, & Mokro, 2009), and the items of these scales could not cover all possible performances of sadism, such as dirty words and urination (Marshall & Hucker, 2006). It is a complete sorting of sadomasochistic performance that researchers must accomplish to construct a corresponding framework for rating scales, containing items for both sadists and masochists and for both pathological and non‐pathological sadomasochists who may present different sadomasochistic trends.

Existing research efforts have focused on etiological pathways of sadomasochism, potential mental health and sexual function issues, and sadomasochistic behaviors and fantasies (Botta et al., 2019; Brown et al., 2019; De Neef et al., 2019). However, most of these studies were descriptive, and very few focused on the underlying driving processes, on establishing comprehensive etiological pathways for the development of sadomasochistic interests (Brown et al., 2019; De Neef et al., 2019), or on constructing a categorization framework of sadomasochistic fantasies and behaviors.

Problems in practice mentioned above imply a lack of comprehensive understanding of the driving process and performance of sadomasochism despite increasing studies on related topics.

### Driving process of sadomasochism

Psychoanalytic theory has persisted regarding the driving process of paraphilia. It is believed that sadomasochism is anchored in childhood experiences, especially trauma (Freud, 2016). Thus, growth history is one of the main themes of etiological research.

Hall (2014) found that masochistic reactions were shaped by early events that left individuals feeling helpless, needy, and unable to defend themselves in the hands of powerful caregivers. L. Sun (2003) found that female sadomasochists experienced guilt due to early love for their fathers and tension with their mothers. Other studies have also reported sadomasochists' negative experiences, including the loss of mother since childhood (Liu et al., 1997), frustrating incidents (Zhao, 2014), crime history or imprisonment (Nitschke, Osterheider, & Mokros, 2009; Nitschke, Ottermann, & Mokro, 2009; Richters et al., 2008; Zhao, 2014), being neglected by parents or caregivers (Nitschke, Osterheider, & Mokros, 2009; Nitschke, Ottermann, & Mokro, 2009), and abuse (Abrams et al., 2022; Chen et al., 2015; Li et al., 2019). Previous studies have shown that sadomasochists are more likely to experience sexual trauma than are ordinary people (e.g., Chen et al., 2015; Lu, 2004; Yost & Hunter, 2012), whereas other studies have proven the contrary (e.g., Hillier, 2019; Myers et al., 2010; Richters et al., 2008).

Several studies have investigated the educational experiences of sadomasochists, showing that they may have less (Zhao, 2014) or more education (Fennel, 2018; Pascoal et al., 2015; Richters et al., 2008). Studies have found that sadomasochists' growth experiences also include cruelty to animals (Stupperich & Strack, 2016) and having earlier sexual experiences (Oliveira Júnior & Abdo, 2010).

These results offer divergent support for the psychoanalytic theory. The first aim of this study was to test the psychoanalytic theory and explore empirical support for pathways of pathological or non‐pathological sadomasochism and the existing etiological model of sadomasochism.

### Performance of sadomasochism

Regarding sadomasochistic behaviors, Richters et al. (2008) found that sadomasochists were significantly more likely to have: had vaginal, oral, or anal sex; been nonexclusive in a stable relationship; had phone sex; deliberately visited an Internet sex site; used a sex toy; had group sex; and engaged in digital anal stimulation, fisting, or rimming. Sadomasochists engage not only in sadomasochistic sex but also in other types of sexual behaviors (Pascoal et al., 2015). A study found that most sadomasochists had non‐BDSM sex as well, with only 11.2% indicating that BDSM was their only form of sexual activity (Nordling et al., 2006). Regarding the diverse sadomasochistic behaviors, 58.9% of male and 54.4% of female practitioners listed bondage as one of their favorite BDSM activities, 73.8% of men and 90.4% of women favored physical pain, and 56.7% of men and 59.2% of women enjoyed humiliation (Botta et al., 2019). In addition, studies have pointed out that sadomasochists exhibit behavioral characteristics such as violent abuse, bondage, aggression (Robertson & Knight, 2014), and antisocial punishment (Pfattheicher & Schindler, 2015). Researchers have found that sadomasochistic behaviors are diverse and intrinsically linked; however, they can be categorized. For instance, Alison et al. (2001) revealed four domains of the behavior: hypermasculinity (e.g., rimming, dildo, cock binding), administration of pain (e.g., spanking, caning, hot wax), humiliation (e.g., verbal humiliation, gagging, face slapping), and physical restrictions (e.g., bonding, chains, wrestling). Existing studies have pointed out that power, rather than giving and receiving pain, is at the core of sadomasochism (Bai & Luo, 2017; Cross & Matheson, 2006; Faccio et al., 2020). Sadomasochists gain sexual pleasure through unequal power relationships (Bai & Luo, 2017). This unequal power relationship may be implemented as a diverse form of sadomasochistic behavior.

As for sadomasochistic fantasies, there is diverse content, such as fetishes (Weiss, 2015), domination, submission, and spanking (Pascoal et al., 2015), quite similar to sadomasochistic behaviors. Among these fantasies, fetishism was of interest to 40.4% of men and 47.9% of women (Joyal & Carpentier, 2016). Women (64.6%) reported fantasizing about being dominated significantly more frequently than men (53.5%), whereas men (59.6%) reported fantasizing about dominating someone significantly more frequently than women (46.7%) (Joyal et al., 2015). One study found that the prevalence of sadomasochistic fantasies was higher than that of sadomasochist behaviors. While interest and fantasy rates are quite high, engagement in BDSM is lower, usually around 20%–30%, showing that having these sexual fantasies does not necessarily mean enacting them (A. Brown et al., 2019). Besides, sexual fantasies exist not only in sadomasochists but also in the general population. For example, in a sample of Canadian university students, 65% had fantasies about being tied up, and 62% had fantasies about tying up a partner (Renaud & Byers, 1999).

Diagnosis and scaling aim to effectively categorize sadomasochistic behaviors and fantasies to evaluate sadomasochistic tendencies, traits, or levels of sadists, masochists, and even switches. Diagnosis criteria and scales should be available for both paraphilic and kinksters. Previous studies have rarely constructed rating scales based on information collected on sadomasochistic behaviors and fantasies, nor have they developed or revised diagnostic criteria or a corresponding treatment program according to the correlation between growth history and sadomasochistic behaviors and fantasies. The second aim of this study was to collect detailed information on sadomasochistic behaviors and fantasies and test their correlation with growth experience.

Studies in Western countries have continuously shown interest in sadomasochism or BDSM (e.g., Abrams et al., 2022; Paarnio et al., 2022; Pascoal et al., 2015; Richters et al., 2008; Wismeijer & Assen, 2013), most of which have tried to collect participants' growth history using quantitative surveys. However, research on this topic in China is scarce. With the development of Internet technology and online communities, an increasing and younger Chinese population is engaging in sadomasochism. Younger sadomasochists may especially face a high risk of being hurt physically or mentally, whether they are pathological or non‐pathological. Cases related to lethal sexual abuse have aroused confusion and bias about sadomasochism in society. These are calling for research focusing on Chinese sadomasochists. This study uses qualitative methods to provide rich information that would not be obtained using quantitative methods. We aim to fill the gaps in previous studies on sadomasochistic driving processes and performance based on Chinese samples to help with judicial disposition, clinical intervention, and mental health services.

## METHOD

This study aims: (1) to describe Chinese sadomasochists' growth experience; (2) to describe Chinese sadomasochistic behaviors and fantasies; and (3) to explore the correlation between Chinese sadomasochists' growth experience and their sadomasochistic behaviors and fantasies, and build a rational model.

### Sample

Fifty‐one Chinese sadomasochists from a Chinese sadomasochistic website formed a convenience sample (see Table 1 for the sample characteristics). We published information about this study via an online link until the planned sample size (*N* = 50) was reached. The link presented the purpose and contact information to anyone who self‐identified as a sadomasochist and was willing to participate in the interviews. The link could be shared online. We decided on the planned sample size according to Britten's (1995) suggestions. However, scheduling issues resulted in a complete sample of 51 sadomasochists.

**TABLE 1:** Demographic information of the sample.

	*N*	Percentage (%)
Gender		
Male	49	96.08
Female	2	3.92
Age		
20–29	18	35.29
30–39	30	58.82
40–49	3	5.88
*M* ± *SD*	31.10 ± 4.20	
Role		
Sadist	28	54.9
Masochist	22	43.14
Switch	1	1.96
Education		
Less than undergraduate		
High school	1	1.96
Technical secondary school	2	3.92
Undergraduate		
Bachelor	28	54.9
Associates	1	1.96
More than undergraduate		
Master's	12	23.53
Doctorate	6	11.76
Inconvenient to inform	1	1.96
Regular partner		
Yes		
Married or engaged	17	33.33
In a romantic relationship	4	7.84
In a sadomasochistic relationship	1	1.96
Married and in a sadomasochistic relationship	1	1.96
No		
Single	22	43.14
Divorced	3	5.88
Inconvenient to inform	3	5.88
Occupation	Staff, doctors, salesmen, students, clerks, sports agents, photographers, business owners, typesetters, teachers, researchers, managers, civil servants, workers, engineers, chefs, cashiers, programmers	
Industry	Finance, culture, foreign trade, software, building materials, environmental protection, Internet, E‐commerce, catering, IT, administration	

### Interview

A semi‐structured interview was developed based on previous studies on sadomasochistic driving processes and performance, including questions on growth experiences, sadomasochistic behaviors, and fantasies. Furthermore, a question concerning whether the interviewees associated their sadomasochistic interests with their experiences was added to collect their viewpoints. Interview themes and questions were developed and finalized through discussions between the researchers and three interviewees, which provided feedback on intelligibility and answerability.

The final interview consisted of five questions (see Supplementary Material 1 in Data S1), followed by follow‐up questions that depended on the responses. The interviewees were prompted to narrow the memory range they would search for the question by adding prompting words to the original questions.

### Procedure

Interviews were conducted between June and August 2019. We presented an informed consent form to each interviewee before the interview and started the interviews only after obtaining their consent and willingness to be participants in this study. Every interviewee was told that they could refuse to answer questions or withdraw from the interview at any time for any reason. All interviews were conducted online via text, voice messages, and voice calls. The interviews lasted 20 to 40 minutes. Three interviewees declined to provide information about their education, occupation, and relationship status, but none withdrew from the interview or retracted consent.

### Data analyses

The interviews were transcribed verbatim and anonymized in the recorded documents. All interview records were imported individually into Nvivo 12 and were coded and analyzed drawing on Corbin and Strauss's (2015) coding system and D. Liu et al.'s (2019) element analysis path. After coding, comparing, discussing, revising, and reaching agreement, we formed a coding manual. The final kappa coefficients of the codes were 0.96–1.00. To present specifically how the coding process led to the coding results and formed the three‐level coding system, the primary, secondary, and tertiary coding process will be shown in the Results section. Cluster analysis by code similarity and model construction, the two function modules of Nvivo 12 designed for testing and presenting relationships among concepts, were used to present the relationship between the interviewees' growth experiences and their sadomasochistic behaviors and fantasies.

According to D. Liu et al. (2019), the quantity of a node's reference points (RPs) represents the typicalness of its content, indicating how the interviewees were concerned about the issue. The amounts of all the nodes' RPs from each interview record were input to SPSS 23 to perform difference test and correlation analysis. The difference test adopted the chi‐square test on positive and negative events, traditional sex and oral/anal sex, and supporting and not supporting the correlation between growth experience and sadomasochistic interests. Correlation analysis on the relationship between growth experiences and sadomasochistic behaviors and fantasies was conducted using the Pearson product–moment correlation coefficient.

## RESULTS

### Overview

Table 1 presents the demographic characteristics of the participants.

There were 25 interviews conducted using voice calls or voice messages, and there were 17 and nine interviews conducted using text messages and voice plus text messages, respectively. We transcribed all the interview materials into interview record documents containing 32,000 Chinese characters.

#### Primary coding

“Primary coding” refers to Corbin and Strauss's (2015) open coding, which breaks up the data, sketches out concepts to represent the original data blocks, and describes these concepts in terms of attributes and dimensions. After reading the interview records repeatedly, we began coding line‐by‐line and determined the RPs. The interview questions originally divided the interview records into parts with different topics, but they required supplementary sorting and categorizing. To explore any possible experience related to sadomasochistic interests and any possible performance of sadomasochism, the primary coding process obtained all the interviewees' descriptions except for meaningless colloquialisms. We initially formed the following categories: demographic data, impressive experience in memory (IEM), sadomasochistic behaviors and fantasies (SBF), and the relationship between sadomasochistic interest and experience (RBSE).

#### Secondary coding

“Secondary coding” refers to Corbin and Strauss's (2015) axial encoding, which is the interpenetration or connection of concepts. For comparison with previous findings and exploration of the correlation between IEM and SBF, the secondary coding paradigm was derived from the “contexts,” “contingencies,” “consequences,” and “covariances” from “the six C' s” coding family (Corbin, 1998), from which elements were further extracted.

- Secondary coding based on the “IEM” category initially formed in the primary coding stage was as follows. In this part, the “IEM” category was defined by this study as the “contingencies,” below which this study extracted the whole incident and the time, people, things, behaviors, and feelings elements from the incidents. These elements were defined as hints of the “covariances” of IEM and SBF.
  1. Interviewees' description of “incident” was coded with the whole paragraph of the “incident” as a unit, such as:

“It was… my neighbor fellow seemed to have stolen his sister's underwear, and he was very turned on. I remember such things.”

2 Interviewees' description of “time” was coded with the sentence of the time period or time point, such as:

“When I was in middle school… when I was in middle school… when I was in the third year of junior high…”

3 Interviewees' description of “people” was coded with the nouns and pronouns as a unit, such as:

“I feel like my **parents** influenced me to like this thing. I lived with my **parents** when I was a child, and I saw **them** having sex. I felt curious about this, and I would think about it at night.”

For this paragraph, we created a node “parent” under the node “experience,” and coded the two “parents” and “them” from the text into the node “parent” (if the node “parent” has been created already, we directly coded “parent(s)” and “them” into that node).

4 For the things mentioned by the interviewees, including items and body parts, we coded the noun as a unit (together with attributives, if there were any), except for those representing time or people, such as:

“memory,” “unwashed panties,” “foot.”

5 For the behaviors and actions mentioned by the interviewees, we coded the verb as a unit:

“stole,” “wears,” “have seen.”

6 For the feelings and emotions mentioned by the interviewees, we coded the adjective, adverb, or noun as a unit, except for those that had already been coded as attributives with their qualifying nouns, such as:

“excited,” “elegant,” “severely,” “very clean.”

7 If the interviewee did not mention any impressive growth experience, we coded the corresponding expression into the sub‐node “none” under the node “experience,” such as:

“no,” “no impressed experience,” “can't remember.”

- The secondary coding for the “SBF” category initially formed in the primary coding stage was as follows. In this part, the “SBF” category was defined by this study as the “consequences,” below which this study extracted the partners, scene, and play elements from the descriptions. These elements were defined as hints of the “covariances” of IEM and SBF.
  1. The interviewees' descriptions of their sadomasochistic partners were coded with phrases or sentences as units:

“my discipline object,” “has a good figure,” “teacher.”

2 The interviewees' descriptions of the scenes in which they engaged in sadomasochism, defined as the “context” from “the six C' s”, was coded with a phrase or sentence as a unit:

“in the wild,” “in the hotel,” “online.”

3 The interviewees' descriptions of sadomasochistic play were coded with play as a unit:

“bondage,” “kneeling at the queen's feet like a slave,” “humiliate,” “role‐playing.”

#### Tertiary coding

“Tertiary coding” refers to Corbin and Strauss's (2015) integration, which involves linking other categories around a core category, and refining and arranging the corresponding theoretical constructs. Tertiary coding results in several sub‐nodes for each category.

There were four parent nodes of the “IEM” category: “IEM‐incident (type) [IEM‐I(T)],” “IEM‐incident (nature) [IEM‐I(N)],” “IEM‐people (IEM‐P),” “IEM‐time (IEM‐T),” and “IEM‐words (IEM‐W).” Ten child nodes of “IEM‐I(T)” were integrated from the nodes in the secondary coding stage, among which three contained grandchild nodes. According to the Life Event Scale's (LEF's) distinction of positive and negative events (c.f., Xu et al., 1996), three child nodes of “IEM‐I(N)” were integrated. Nine and three child nodes of “IEM‐P” and “IEM‐T” were integrated, respectively. Additionally, two child nodes of “IEM‐W” were integrated, among which “related to sadomasochism” had five grandchild nodes.

There were three parent nodes in the “SBF” category: “SBF‐play (SBF‐PL),” “SBF‐partner (SBF‐PTN),” and “SBF‐scene (SBF‐SC).” According to the five categories of sadomasochistic play developed by Chinese sadomasochists, five child nodes of “SBF‐PL” were integrated, among which “sex” had two grandchild nodes. Five child nodes of “SBF‐PTN” were integrated.

There was one parent node of the “RBSE” category, and three child nodes were integrated.

Thus, this study formed a three‐level coding system for the interviewees' impressive growth experiences, their sexual behaviors and fantasies, and the relationship between the two, as shown in Tables 2, 3, 4.

**TABLE 2:** Three‐level coding system of interview records (IEM‐I section).

Level 3	Level 2	Level 1
IEM‐I(T) (what)	Interested in sadomasochism things since childhood	–
	Family upbringing	Lack of love
		Parents' beating and scolding
		Educated according to a certain standard
		Parents' consolation
	Parents' marriage problems	–
	Watched literary works or websites	Books
		Films and television shows
		Websites
	Noticed female image	–
	Negative experiences during school	–
	Sexual experience	Masturbation
		Others' sex
		Own sex
		Intimacy with mother
		Fetish
		Sex games
	Personality trait since childhood	
	Other	–
	None	–
IEM‐I(N) (how)	–	Positive event
	–	Neutral event
	–	Negative event

Abbreviations: IEM, impressive experience memory; IEM‐I(T), IEM‐incident (type); IEM‐I(N), IEM‐incident (nature).

**TABLE 3:** Three‐level coding system of interview records (IEM‐P, T, W sections).

Level 3	Level 2	Level 1
IEM‐P (who)	–	Parent
	–	Teacher
	–	Character
	–	Childhood playmate
	–	Classmate
	–	Sex partner
	–	Relative
	–	“Sister” unrelated by blood
	–	Other
IEM‐T (when)	–	Infancy and childhood
	–	Adolescence
	–	Youth
IEM‐W (element)	Unrelated to sadomasochism	–
	Related to sadomasochism	Spirit
		Sex
		Punishment
		Canine
		Excretion

Abbreviations: IEM, impressive experience memory; IEM‐P, IEM‐people; IEM‐T, IEM‐time; IEM‐W, IEM‐words.

**TABLE 4:** Three‐level coding system of the interview records (SBF and RBSE sections).

Level 3	Level 2	Level 1
SBF‐PL	Punishment	–
	Canine	–
	Sex	Traditional sex
		Oral/Anal
	Spirit	–
	Excretion	–
SBF‐PTN	–	Personality and temperament
	–	Relationship mode
	–	Appearance
	–	Personal identity and occupation
	–	Age
SBF‐SC	–	–
RBSE	–	Related
	–	Unrelated
	–	Unclear

Abbreviations: RBSE, relationship between sadomasochistic interest and experience; SBF, sadomasochist behaviors and fantasies; SBF‐PL, SBF‐play; SBF‐PTN, SBF‐partner; SBF‐SC, SBF‐scene.

### Interviewees' impressive experience in memory

Many researchers advocate using psychoanalytic theories to help sadomasochistic clients (e.g., Chen & Cui, 2005; Grossman, 2015), therefore, understanding sadomasochists' experiences is significant in professional work. This section analyzes interviewees' memorable experiences from different levels and perspectives. At the macro level, we analyzed the whole incident from their experiences, whereas at the micro level, we analyzed the incident elements.

From the IEM part of the interview records, 142 + 131 + 176 + 86 + 908 RPs were extracted in the secondary coding stage, and 14 + 3 + 9 + 3 + 5 Level 1 and 10 + 0 + 0 + 0 + 2 Level 2 codes were formed in the tertiary stage.

#### IEM‐I

According to the number of Level 2 code RPs sorted from highest to lowest, we formed the node tree of the “incident” from IEM, as shown in Tables 5 and 6.

**TABLE 5:** Node tree of IEM incident (type) code.

Level 2	Level 1	RPs (source)	Interview example	Supplementing explanation	RP proportion (%)
Sexual experience	Own sex (13)	39 (15)	“While I just graduated from school and started my career, I tried to humiliate and discipline a girl. That girl, I disciplined her. I was 20 years old at that time. I beat her and scolded her.”	The interviewee had sex personally.	9.15
	Masturbation (8)		“When I was in elementary school, I learned to masturbate.”	The interviewee tried masturbation.	5.63
	Others' sex (8)		“I lived with my parents when I was a kid so I could see them having sex and felt so curious about it, and I would think about it at night.”	The interviewee witnessed other people's sex.	5.63
	Fetish (4)		“In high school, I once smelled my sister's panties in the toilet. I fell in love with the panties' kind of smell and turned on.”	The interviewee was turned on by an inanimate object.	2.82
	Intimacy with mother (3)		“I remember when I was very young, my mother rubbed her breasts against me, but I thought it was my mother cuddling me.”	The interviewee was physically close with his mother.	2.11
	Sex games (3)		“I remember playing with the kids when I was very young, and then, just for fun, we played the game of scratching each other's genitals.”	The interviewee had fun by playing games related to sex.	2.11
Family upbringing	Educated according to a certain standard (11)	28 (17)	“When I was young, my parents often taught me to control myself and handle the situation well, etc.”	The interviewee was educated to be rational and strong.	7.75
	Parents' beating and scolding (10)		“One time, I should be very young then, but I'm not sure how old I was, maybe three or 4 years old. I also forgot my mistakes, and I only remember my mother hitting me all over with a clothes hanger. I've forgotten the process of beating, either. What impressed me more was that after the fight, my parents took off my pants and put medicine on the wound because there were many bloodstains, one after another. I still remember that my legs were tiny, white, and tender. The blood on it was shocking, and my parents raised my legs to give me medicine. I could only remember this scene. As for how the other things happened and what happened later, I forgot.”	The interviewee was hit and hurt so bad by her parents.	7.04
	Lack of love (6)		“When I was a child, I lacked love. My parents rarely cared about me, and I felt little family warmth.”	The interviewee barely gained love from the caregivers.	4.23
	Parents' consolation (1)		“They comforted me and told me my classmates' gossip will not affect me.”	The interviewee gained consolation from parents in a hard situation.	0.70
Watched literary works or websites	Books (10)	20 (12)	“After I saw some pictures and articles in English, I was absorbed and excited by the things related to bondage in it.”	The interviewee read from written material.	7.04
	Films and television shows (8)		“As for the films and television shows, I could remember some soap operas from Hong Kong, telling stories of a stepfather and his daughter. They had coitus with each other and then lay down and had a conversation. The girl seemed so slutty. Later, I was so impressed by the movie *Hana to Hebi*, which shows tied up and humiliated women's unprecedented sexual appeal. I watched so many of Dan Oniroku's works in high school.”	The interviewee watched films and television shows.	5.63
	Websites (2)		“When I was in high school, I accidentally discovered the sadomasochism community and joined it.”	The interviewee scanned a website.	1.41
Interested in sadomasochism things since childhood	–	13 (8)	“I remember when I was little, 7 or 8 years old, I liked hiding in mosquito nets. The feeling of hiding up when playing hide‐and‐seek was so appealing to me. And I liked to be rolled into the quilt. I feel that it may have been an initial subconsciousness of bondage.”	The interviewee was absorbed into the feelings of being bound up since little.	9.15
Notice female image	–	10 (6)	“When I was in middle school, yeah, when I was in middle school, when I was in the third year of junior high school, there were senior classmates… senior classmates at that time. There was a class in our middle school called the vocational high class, educating all the flight attendants in the future and the hotel's senior waiter. They look so elegant and beautiful, wearing high‐heels and professional clothes, wandering around the campus, as can be often seen.”	The interviewee found himself appreciating the female senior classmates' good looks.	7.04
None	–	11 (11)	“No. My growth experience is very ordinary.”	The interviewee did not report any impressive incidents.	7.75
Negative experiences during school	–	8 (2)	“When I was young, I was average in all aspects and did not get as much attention as my classmates. I felt everyone was getting attention, but I was unknown and did not get everyone's approval.”	The interviewee wasn't cared about much in the campus life as he wished.	5.63
Other	–	8 (6)	“I played with the playmates, and we ate together when I was a kid.”	The interviewee's description of the incident was difficult to categorized to any of the nodes.	5.63
Personality trait since childhood	–	3 (3)	“My growth experience is nothing special. I am a domineering Sagittarius man. I have shown a desire to conquer since I was a child.”	The interviewee behaved domineeringly since he could remember.	2.11
Parents' marriage problems	–	2 (2)	“It's my parents getting divorced. I only remember them getting divorced.”	The interviewee's parents split up.	1.41

Abbreviations: IEM, impressive experience memory; RP, reference point.

**TABLE 6:** Node tree of IEM‐incident (nature) code.

Level 1	RPs (source)	Interview example	Supplementing explanation	RP proportion (%)
Neutral event	58 (25)	“I played with the playmates, and we ate together when I was a kid.”	The incident was neither negative nor positive to the interviewee.	44.27
Negative event	39 (17)	“When I was in college, I was hanging out with a male friend and a female friend. I liked that girl, but I accidentally saw them hugging each other. I'm a lot calmer now, but it's vivid in retrospect. At that time, my mind was blank, my chest was tight, and I felt suffocated. That boy was a master at seducing girls. He knows how to advance, retreat, break girls' defenses, and take advantage. What shatters values is that there's no hope for honest people.”	The incident caused negative emotion of the interviewee.	29.77
Positive event	34 (17)	“That masochist accepts all kinds of tastes, which I especially like and could feel very satisfied from.”	The incident brought positive feelings to the interviewee.	25.95

Abbreviations: IEM, impressive experience memory; RP, reference point.

In Level 2, the node “sexual experience” contains descriptions of sex‐related experiences in memory. The child node “own sex” refers to the interviewees' own sex‐related experiences, which were usually exploratory. They mentioned traditional sexual and sadomasochistic experiences, including active and passive sex. The child node “masturbation” refers to how they satisfied themselves sexually and their sexual fantasies during masturbation. The child node “others' sex” refers to their memories of other people's sexual experience, mainly witnessing family members' or animals' sex. The child node “fetish” refers to sexual arousal caused by objects, and the main items mentioned were underwear and socks. The child node “intimacy with mother” refers to physically intimate contact with one's mother, which was derived from the male interviewees' description. The child node “sex game” refers to the experience of playing sex‐related games with playmates in their early years.

The node “family upbringing” contains the family education experience in memory. The child node “educated according to a certain standard” refers to being trained and disciplined according to a certain standard by parents, within which the interviewees mentioned the ideals of “independence,” “self‐control,” “introspection,” “polite,” “sensible,” and so forth. The child node “parents' beating and scolding,” “lack of love,” and “parents' consolation” are self‐explanatory.

The node “watch literary works or websites” contains the experience of watching various literary and artistic works or accessing related information on the Internet. The child nodes “books,” “film and TV show,” and “website” are self‐explanatory.

The node “interested in sadomasochistic things since childhood” contains the interviewees' interest in sadomasochistic things since they could remember. The node “notice female's image” contains the memory of the interviewees actively or passively noticing the image of women, all of which were from male interviewees, mentioning their senior sisters, mothers, and female relatives. The node “none” means that the interviewee did not mention any impressive experiences in memory. The node “negative experiences during school” contains memories of negative emotions on campus, including being ignored, denied, and ridiculed by teachers or classmates. The node “personality trait since childhood” describes the sadomasochistic characteristics without clear incentives that have existed since the interviewees could remember, including rebellion, desire to conquer, and desire to control. The node “parents' marriage problems” contains memories of the interviewees' parents' divorce or affair.

The nodes “positive event,” “negative event,” and “neutral event” contain the statement of positive, negative, and neutral incidents for the interviewees in terms of their actual feelings.

Table 5 shows that most interviewees (*N* = 17) mentioned the experience of family upbringing, and the RPs accounted for 19.72%, indicating that family upbringing is an important experience for sadomasochists. Among these incidents, the RP of “educated according to a certain standard” was most prevalent (7.75%) followed by “parents' beating and scolding” (7.04%, mentioned by seven interviewees). Parents' consolation was mentioned less, with one RP accounting for 0.7%.

Although only 15 interviewees mentioned sexual experience, the RPs accounted for the majority (27.46%), indicating that sexual experience played a vital role in sadomasochists' growth experience. Among sexual experiences, the interviewees' own experiences were mentioned the most (9.15%).

The experience of watching literary works or websites ranked third. Still, the RP amount of reading sadomasochism‐related books was the same as parents' beating and scolding (7.04%), revealing that reading sadomasochism‐related books also played a role in the sadomasochist growth experience.

Table 6 shows that most interviewees (*N* = 25) mentioned neutral events, followed by negative and positive events, and the RPs accounted for 44.27%, 29.77%, and 25.95%, respectively. The *χ* ^*2*^ analysis was performed on the RPs of positive and negative events nodes, showing no significant difference between the two types of events (*χ* ^*2*^ = 0.342, *p* = .558, *p* > .05). This indicates that sadomasochists do not necessarily experience more negative events than positive ones.

#### IEM‐P

According to the number of Level 1 code RPs sorted from highest to lowest, we formed a node tree of the people from the IEM, as shown in Table 7.

**TABLE 7:** Node tree of IEM‐people code.

Level 1	RPs (source)	Interview example	Supplementing explanation	RP proportion (%)
Parent	63 (17)	“Probably during high school, I woke up in the middle of the night and saw mommy giving daddy a blowjob.”	–	35.80
Relative	29 (7)	“My first sexual experience was with my second aunt.”	A second aunt (远方姑姑) is a sister of the interviewee's father.	16.48
Character	16 (3)	“It seemed to be a Hong Kong TV episode, showing scenes on a bed with probably a daughter and her stepfather hooking up in it.”	They are roles from a TV show.	9.09
Other	15 (5)	“I also fantasize about serving my teacher, helping her and her lover clean up.”	It's people who were not typical enough to create an independent code for.	8.52
Teacher	13 (3)	“During high school, I went to my tutor's home for tutoring.”	It's the interviewee's teacher for shadow education.	7.39
Sex partner	12 (4)	“That masochist can take deep blowjob, spanking, being humiliated, and I really enjoy those feelings.”	It's the interviewee's partner for sexual activities.	6.82
Childhood playmate	11 (6)	“I played and ate with the kids.”	They are fellows the interviewee used to play with during childhood.	6.25
“Sister” unrelated by blood	10 (5)	“The neighbor seemed to have stolen his sister's underwear.”	It's an older peer female.	5.68
Classmate	7 (5)	“At the beginning of my junior high school years, my classmates took me to an Internet café.”	They are students the interviewee used to study with in the same class.	3.98

Abbreviations: IEM, impressive experience memory; RP, reference point.

The node “other relatives” includes relatives other than parents, and the interviewees talked about grandparents, sisters, aunts, and so forth. The node “‘sisters’ unrelated by blood” includes older females of the same generation, and the interviewees mainly talked about girls from the neighborhood, senior sisters, and so forth. The node “other” includes other people, and the interviewees talked about friends, parents' extramarital affairs, neighbors, and so forth.

Table 7 shows that 17 interviewees mentioned “parents,” and these RPs ranked first (35.80%), followed by “other relatives” (16.48%) mentioned by seven interviewees.

#### IEM‐T

According to the number of Level 1 code RPs sorted from highest to lowest, we formed the node tree of the time of the IEM, as shown in Table 8.

**TABLE 8:** Node tree of IEM‐time.

Level 1	RPs (source)	Interview example	Supplementing explanation	RP proportion (%)
Infancy and childhood	56 (30)	“When I was in primary school”	This time period equals about 12 years old and younger (Grade 6 and before).	65.12
Adolescence	24 (14)	“When I was in middle school”	This time period equals about 13 to 18 years old (Grade 7 to 12).	27.91
Youth	6 (5)	“When I was in college”	This time period equals about 19 to 22 years old (undergraduate education).	6.96

Abbreviations: IEM, impressive experience memory; RP, reference point.

Table 8 shows that 30 interviewees mentioned infancy and childhood, and these RPs (65.12%) ranked first, followed by the RPs of the node “adolescence” (27.91%) mentioned by 14 interviewees, which probably indicates that the incidents happening during infancy and childhood were more impressive compared with the ones during other periods.

#### IEM‐W

According to the number of Level 2 code RPs sorted from highest to lowest, we formed a node tree of the words from the description of IEMs, as shown in Table 9.

**TABLE 9:** Node tree of IEM‐words.

Level 2	Level 1	RPs (source)	Interview example	Supplementing explanation	RP proportion (%)
Unrelated to sadomasochism	–	542 (45)	“When I was in college, I went out to play with a friend and a girl. I liked that girl, but I accidentally saw them hugging each other. I'm calmer now, but I remember it vividly. At that time, my mind was blank, my chest was tight, and I felt suffocated.” “The neighbor friend seemed to have stolen his sister's underwear, and then he was very turned on. I remember such a thing.”	These are neutral words.	59.69
Related to sadomasochism	Spirit (128)	366 (37)	“Later, I was so impressed by the movie *Hana to Hebi*, which shows tied up and humiliated women's unprecedented sexual appeal.”	The feeling “so impressed” was caused by the humiliated women. “Humiliated” is an emotion caused by being tied up.	14.10
	Sex (103)		“Later, I was so impressed by the movie *Hana to Hebi*, which shows tied up and humiliated women's unprecedented sexual appeal.”	“Sexual appeal” is mainly a sex symbol of the women.	11.34
	Punishment (63)		“The most impressive is that after scolding me, my parents took off my pants and put medicine on the wound because there were a lot of bloodstains.”	These words are related to pain and injury.	6.94
	Canine (39)		“They look so elegant and beautiful, wearing high‐heels and professional clothes, wandering around the campus, as can be often seen.”	Socks and shoes, including “high‐heels” are typical symbol of fetish related to smell and pets.	4.30
	Excretion (15)		“When I was a pupil, I saw people being drenched in urine on TV.”	Excrement is the key point of “being drenched in urine.”	1.65

Abbreviations: IEM, impressive experience memory; RP, reference point.

In Level 2, the node “unrelated to sadomasochism” contains words without any meaning of sadomasochism, such as “brain,” “remember,” and “blank.” The node “related to sadomasochism” contains words with sadomasochistic meanings, such as “bondage,” “sexual attraction,” and “shame.” We utilized the categorization of sadomasochistic play, developed alongside Chinese sadomasochism culture, to integrate the nouns, verbs, adjectives, and adverbs coded during the secondary coding into “spirit,” “sex,” “punishment,” “canine,” and “excretion.” The child node “spirit” refers to words related to dominance/submission (D/S), gender identity, or role‐play, which emphasize one's cognition, emotion, and will. “Sex” refers to the words associated with sex. “Punishment” refers to words related to restrictions, pain, injury, or death. “Canine” refers to words related to animals and their habits. For example, they live by relying on smell, and humans rear them. “Excretion” refers to words associated with excrement and excretory organs.

Table 9 shows that the words unrelated to sadomasochism ranked first (59.69%) in Level 2. In Level 1, the RPs of the node “spirit” ranked first (14.10%), followed by “sex” (11.34%), and “excretion” (1.65%) ranked last.

We used this part of the coding results as the data source for correlation analysis with the nodes of the interviewees' sadomasochistic behaviors and fantasies.

### Interviewees' sadomasochistic behaviors and fantasies

Understanding sadomasochism's specific manifestation is necessary for professional work to build basic concepts of sadomasochistic clients' sexual behaviors and fantasies even when they do not realize they have these sexual interests. For example, some people have an urge to tie up the limbs of the opposite sex, accompanied by sexual arousal. Still, they might not think they have sadomasochistic interests because it seems that they “do not hurt people and do not want to have sex with the concerned person either.” Therefore, a more comprehensive and in‐depth analysis of sadomasochistic performance is necessary.

This section analyzes the interviewees' sadomasochistic behaviors and fantasies. There is no clear boundary between sadomasochistic behavior and sadomasochistic fantasy. For example, it could not be said whether a sadomasochistic couple playing a daddy–daughter scene, where the “daddy” sexually punishes the “daughter,” is exhibiting a sadomasochistic behavior or a sadomasochistic fantasy. Thus, we combined the two as one category when analyzing them. Our analysis extracted three elements from them: play, partners, and scenes. This helped us understand what they have done, who their partners were, and where.

From the SBF part of the interview records, 424 + 147 RPs were extracted in the secondary coding stage, and 2 + 5 codes of Level 1 and 5 + 0 of Level 2 were formed in the tertiary stage.

#### SBF‐PL

According to the number of Level 2 code RPs sorted from highest to lowest, we formed the node tree of coding with the play of SBF as a unit, as shown in Table 10.

**TABLE 10:** Node tree of SBF‐play.

Level 2	Level 1	RPs (source)	Interview example	Supplementing explanation	RP proportion (%)
Spirit	–	152 (46)	“Well, kneel at the Queen's feet like a slave.”	The relationship between Queen and slave is a D/S relationship.	35.85
Punishment	–	98 (33)	“Needle play. I've been playing for 9 years.”	Needles bring injury and pain for participants.	23.11
Sex	Oral/Anal (18)	78 (34)	“She put her feet out to play with my genitals and talk to me simultaneously.”	The sex organ was stimulated.	18.40
	Traditional sex (10)				
Excreta	–	54 (11)	“I drink her pee and lick her ass.”	Excrement and excretory organs are involved.	12.74
Canine	–	42 (23)	“Put a collar on him, drive to the field, and then take him for a walk.”	The “him” was treated as a pet.	9.91

Abbreviations: RP, reference point; SBF, sadomasochist behaviors and fantasies.

**FIGURE 1:**
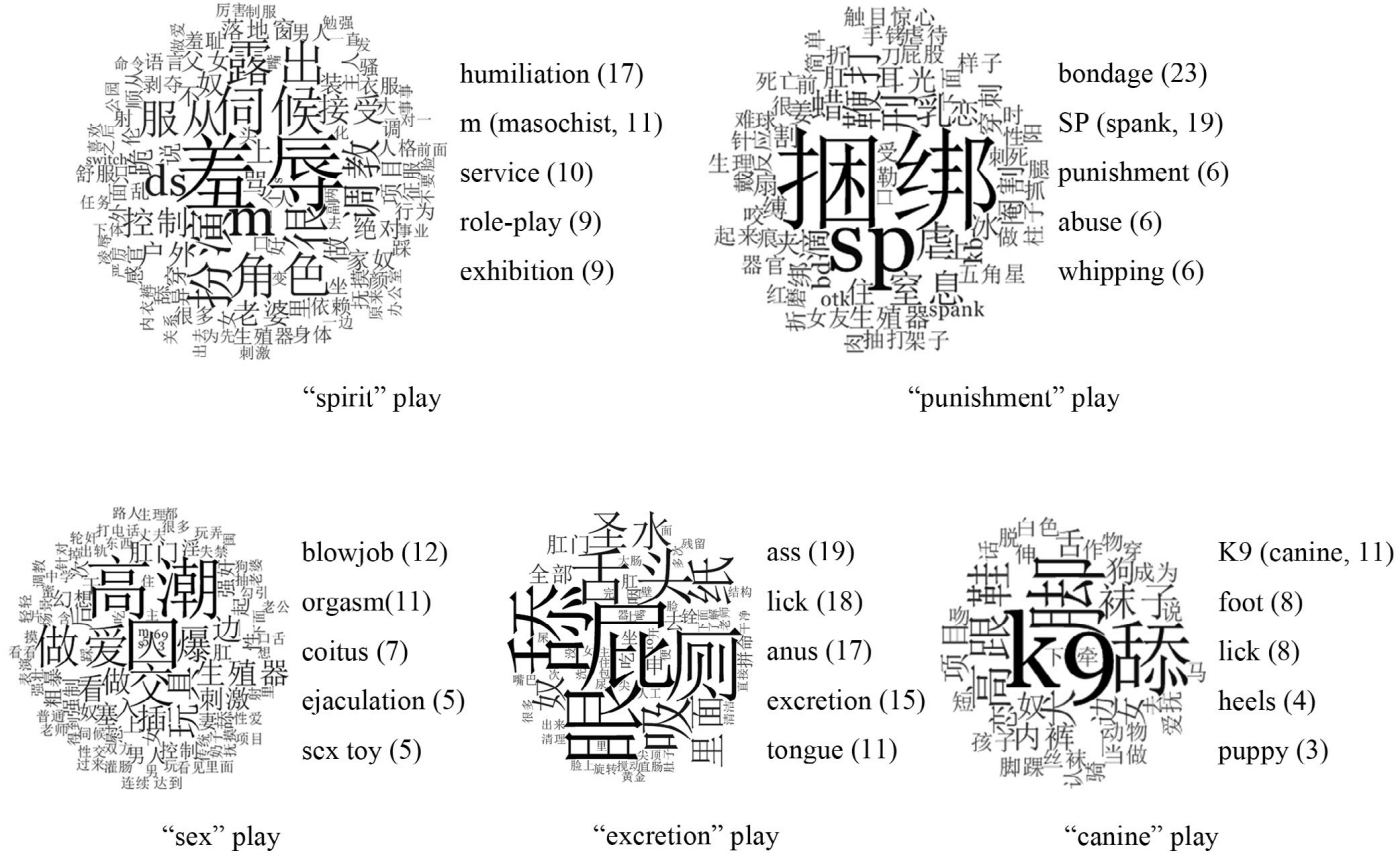
Word cloud of each type of play.

The node “spirit” refers to play related to D/S, gender identity, and role‐play. It emphasized the play's influence on sadomasochists' cognition (e.g., “I belong to my partner”), emotion (e.g., “I feel humiliated”), and will (e.g., “I will take care of my partner as my ‘little sister’”). As psychology is called “*Xin Li Xue* (心理学)” in Chinese, Chinese sadomasochists take “Xin” as the human's organ of cognition, emotion, and will, which was translated into “spirit” in this study. The node “punishment” refers to play related to restraint, pain, injury, or death, emphasizing the play's similarities to punishments (刑) in ancient China (such as the bondage and torture of prisoners). Unlike the types of “spirit” play, which mainly mentally influence sadomasochists, the types of “punishment” play mostly cause physical pain. The node “sex” (性) refers to play related to sexual arousal and satisfaction, emphasizing the direct connection from the play to sexual arousal and satisfaction (such as direct stimulation of the sexual organs). Unlike the types of “spirit” play that mainly cause mental influence and types of “punishment” play that mainly cause physical pain, “sex” play means acting on sexual organs and directly leading to sexual arousal. Child nodes of “sex” are “traditional sex” and “oral and anal sex.” The node “canine” (犬) refers to play related to animals and their habits, as mentioned above. It emphasizes the similarities between a masochistic role and a dog (e.g., licking and crawling), as well as the similarities between a sadistic role and a dog keeper (e.g., feeding, walking a dog). Unlike the dominance and submission in the “spirit” play, the “canine” play emphasizes the doggy appearance of the masochist and the sadist's “training” and “feeding” the masochist. The node “excretion” (厕) refers to play related to excrement and excretory organs, emphasizing the unclean characteristics of the play (e.g., bathing with excrement). Each type of play is presented explicitly by a word cloud, which contains diverse Chinese expressions of the play, as shown in Figure 1. We decided to present the original Chinese word clouds to ensure the faithfulness of the interviewees' descriptions. However, it was important to list the top five most frequent words and translate them into English for the readers according to the interview records.

Table 10 shows that the RPs of “spirit” play (35.84%) ranked first, followed by the “punishment” play (23.11%), while “canine” play (9.91%) ranked last.

The *χ* ^*2*^ analysis was performed on the RPs of “traditional sex” and “oral and anal sex” nodes, showing there was no significant difference between the two (*χ* ^*2*^ = 2.286, *p* = .131), indicating that the interviewees equally preferred the two.

#### SBF‐PTN

According to the number of Level 1 code RPs ranked from highest to lowest, the node tree of coding with the statements on the sadomasochistic partners of the interviewees was formed, as shown in Table 11.

**TABLE 11:** Node tree of SBF‐parent.

Level 1	RPs (source)	Interview example	Supplementing explanation	RP proportion (%)
Personality and temperament	45 (26)	“I'd like a lovely partner.”	“Lovely” is describing the partner's kind personality.	30.61
Relationship mode	41 (29)	“We'd better be friends.”	“Friends” is describing the friendship between the interviewee and his partner.	27.89
Appearance	28 (15)	“I like a healthy, lively, and slender partner because it would look nice to tie her up.”	“Slender” is describing the partner's body figure.	19.05
Personal identity and occupation	23 (11)	“Then they are all highly educated people, such as professional white collars, or professionals, such as doctors, teachers, bank clerks, etc.”	These are words showing the partner's social identity and career.	15.65
Age	10 (7)	“I'd like a partner who's older than me, at least 30 years old, preferably more than 40 years old.”	These are words describing the partner's age.	6.80

Abbreviations: RP, reference point; SBF, sadomasochist behaviors and fantasies.

The node “personality and temperament” contains descriptions of the sadomasochistic partner's personality and temperament. The node “relationship mode” describes how the sadomasochistic relationship works. The node “appearance” contains the descriptions of the partner's look, figure, dress, and make‐up. The node “personal identity and occupation” contains the descriptions of the partner's professional or social identity. The node “age” contains the descriptions of the partner's age.

Table 11 shows that the partner's personality and temperament were mentioned the most, with these RPs accounting for 30.61%. Regarding the RP sources, most interviewees talked about the sadomasochistic relationship mode (27.89%). The partner's age was mentioned the least (6.80%).

#### SBF‐SC

**FIGURE 2:**
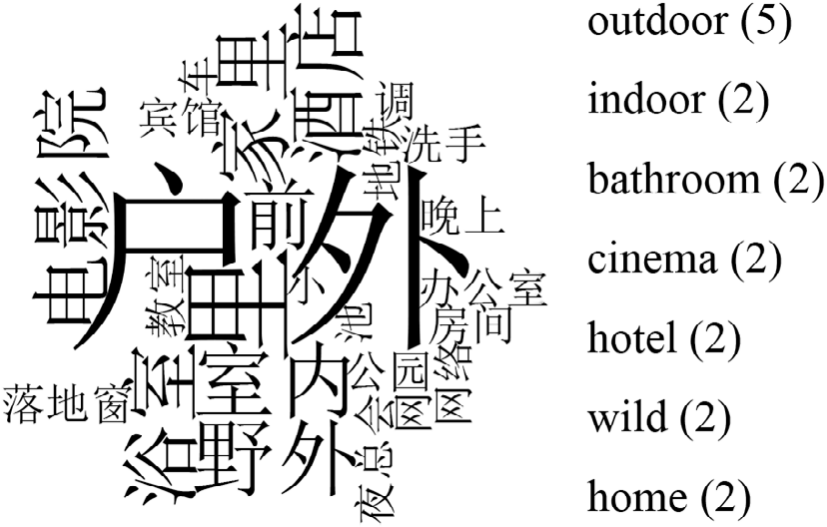
Word cloud of the scene description of interviewees' sadomasochistic behaviors and fantasies.

The content of the node “SBF‐SC” is presented by the word cloud shown in Figure 2.

According to the records, interviewees were more likely to talk about outdoor settings, such as the wild, parks, and subways. They rarely talked about home scenes.

### The correlation between IEM and SBF

This section explores the correlation between interviewees' impressive experience in memory and sadomasochistic behaviors and fantasies from both qualitative and quantitative perspectives. First, we analyzed the interviewees' viewpoints. Second, correlation analysis using SPSS 23 and cluster analysis by code similarity using NVivo 12 were performed to explore the correlation between the two.

#### Interviewees' viewpoints

From the interview record in which interviewees answered the question, “Do you think your sadomasochistic interest is related to your impressive experience in memory or not,” a total of 51 RPs were extracted in the primary coding stage. Three Level 1 and no Level 2 codes were formed in the tertiary coding stage.

According to the number of Level 1 code RPs sorted from highest to lowest, we formed a node tree of interviewees' answers, as shown in Table 12.

**TABLE 12:** Node tree of RBSE.

Level 1	RPs (source)	Interview example	RP proportion (%)
Related	31 (31)	“After I fell in love with sadomasochism when I grew up, I looked back and realized the two are a bit related to each other.”	60.78
Unrelated	11 (11)	“I do not think so.”	21.57
Not clear	9 (9)	“I do not know if they are related to each other.”	17.65

Abbreviations: RBSE, relationship between sadomasochistic behavior and experience; RP, reference point.

Table 12 shows that most of the interviewees believed that their SBF was related to their IEM, with the RPs accounting for 60.78%, followed by those who thought the two were not related (21.57%), and those who were not sure of the correlation (17.65%).

The *χ* ^*2*^ analysis was performed on the RPs of “related” and “unrelated” nodes showing significantly more interviewees believed their SBF was related to their IEM (*χ* ^*2*^ = 9.542, *p =* .002, *p* < .01).

#### Correlation between IEM and SBF

The correlation analysis showed the correlation between how interviewees talked about their impressive experiences during growth and their sadomasochistic behaviors and fantasies during adulthood. This described whether and how elements from past and present life stages were related. A correlation analysis was performed on incidents and play, words and play, and people and partners.

##### Correlation between IEM‐I(T) and SBF‐PL

Correlation analysis was performed on the number of RPs of child and grandchild nodes of “IEM‐I(T)” and “SBF‐PL” nodes, as shown in Table 13.

**TABLE 13:** Correlation between IEM‐I(T) and SBF‐PL.

	Play	Spirit	Punishment	Sex	Canine	Excretion
Sexual experience	0.114	−0.085	−0.069	0.401**	0.297*	−0.016
Own sex	0.032	−0.187	−0.126	0.306*	0.202	0.015
Masturbation	0.143	−0.084	−0.033	0.278*	0.418**	0.016
Other people's sex	−0.072	−0.116	−0.135	0.280*	−0.157	−0.035
Fetish	0.251	0.283*	0.058	0.118	0.340*	0.019
Intimacy with mother	0.015	0.118	−0.199	0.221	0.098	−0.057
Sex games	0.067	−0.146	0.295*	0.054	0.174	−0.044
Family upbringing	0.378**	0.304*	0.152	−0.140	−0.100	0.345*
Educated according to a certain standard	0.098	0.258	0.126	0.026	−0.002	−0.061
Parents' beating and scolding	0.518**	0.263	−0.144	−0.090	−0.195	0.677**
Lack of love	−0.007	−0.019	0.333*	−0.198	−0.033	−0.063
Parents' consolation	0.041	0.001	0.076	−0.045	0.238	−0.032
Watched literary works or websites	−0.069	−0.028	−0.087	0.006	0.171	−0.087
Books	−0.075	0.034	−0.103	−0.105	0.096	−0.054
Films and television shows	−0.041	−0.056	−0.051	0.071	0.146	−0.069
Websites	0.024	−0.092	0.058	0.179	0.106	−0.046
Interested in sadomasochism things since childhood	0.052	−0.102	0.044	−0.024	−0.078	0.125
Notice female image	0.062	−0.012	−0.202	0.490**	0.070	−0.026
Negative experiences during school	0.033	−0.017	0.107	−0.063	0.223	−0.037
Personality trait since childhood	−0.085	−0.152	0.135	−0.030	−0.095	−0.057
Parents' relationship problems	0.129	−0.045	−0.093	0.361**	0.184	0.041

Abbreviations: IEM, impressive experience memory; IEM‐I(T), IEM‐incident(type); SBF, sadomasochist behaviors and fantasies; SBF‐PL, SBF‐play.

** *p* < .01;

* *p* < .05.

It shows that interviewees' mention of sexual experience was moderately correlated with that of sex play (*r* = .401, *p* = .004, *p <* .01) and weakly correlated with that of canine play (*r* = .297, *p* = .034, *p* < .05). Their mention of their own sex was weakly correlated with that of sex play (*r* = .306, *p* = .029, *p* < .05). Interviewees' mention of masturbation was weakly correlated with that of sex play (*r* = .278, *p* = .048, *p* < .05) and moderately correlated with that of canine play (*r* = .418, *p* = .002, *p* < .01). Their mention of other's sex in the past was weakly correlated with that of present sex play (*r* = .280, *p* = .046, *p* < .05), and their mention of fetish was weakly correlated with that of spirit play (*r* = .283, *p* = .044, *p* < .05) and canine play (*r* = .340, *p* = .015, *p* < .05). Their mention of sex games was weakly correlated with that of sex play (*r* = .295, *p* = .036, *p* < .05), and their mention of family upbringing was weakly correlated with that of sadomasochistic play (*r* = .378, *p* = .006, *p* < .01), spirit play (*r* = .304, *p* = .030, *p* < .05), and excretion play (*r* = .345, *p* = .013, *p* < .05). Interviewees' mention of parents' beating and scolding was moderately correlated with that of sadomasochistic play (*r* = .518, *p* = .000, *p* < .001) and strongly correlated with that of excretion play (*r* = .677, *p* = .000, *p* < .001). Their mention of lack of love was weakly correlated with that of punishment play (*r* = .333, *p* = .017, *p <* .05). Interviewees' mention of noticing female images was moderately correlated with their talking about sex play (*r* = .490, *p* = .000, *p* < .001). Their talking about parents' relationship problems was weakly correlated with that about sex play (*r* = .361，*p* = .009，*p* < .01). Among all types of incidents, only parents' beating and scolding during growth accurately predicted excretion play during adulthood.

##### Correlation between IEM‐I(N) and SBF‐PL

Correlation analysis was performed on the RP's number of “IEM‐I(N)” child nodes and “SBF‐PL” and its child nodes, as shown in Table 14.

**TABLE 14:** Correlation between IEM‐I(N) and SBF‐PL.

	Play	Spirit	Punishment	Sex	Canine	Excretion
Positive event	0.138	−0.020	−0.096	0.134	0.357*	0.076
Neutral event	−0.019	−0.067	−0.150	0.260	0.156	−0.064
Negative event	0.315*	0.127	0.082	0.214	0.077	0.202

Abbreviations: IEM, impressive experience memory; IEM‐I(N), IEM‐incident(nature); SBF, sadomasochist behaviors and fantasies; SBF‐PL, SBF‐play.

* *p* < .05.

It shows that interviewees' mention of positive events weakly correlated with that of canine play (*r* = .357, *p* = .010, *p* < .05). The positive events mentioned by the interviewees were mainly regarding the pleasure of exploring sex. Their mention of negative events, mainly the incidents resulting in negative emotions, was weakly correlated with that of sadomasochistic play (*r* = .315, *p* = .024. *p* < .05). There was no significant support for positive or negative events during growth predicting sadomasochistic play during adulthood.

##### Correlation between IEM‐W and SBF‐PL

Correlation analysis was performed on the RPs' number of “IEM‐W” under “related to sadomasochism” and its child nodes and “SBF‐PL” and its child nodes, as shown in Table 15.

**TABLE 15:** Correlation between IEM‐W and SBF‐PL.

	Play	Spirit (*p*)	Punishment (*p*)	Sex (*p*)	Canine (*p*)	Excretion (*p*)
(Words) related to sadomasochism	0.219	0.023	−0.028	0.280*	0.283*	0.096
Spirit (w)	0.202	0.196	0.005	0.101	0.123	0.089
Punishment (w)	0.018	−0.127	0.134	0.169	0.070	−0.056
Sex (w)	0.107	−0.010	−0.183	0.496**	0.292*	−0.042
Canine (w)	0.038	−0.089	0.074	−0.019	0.366**	−0.038
Excretion (w)	0.718**	0.134	−0.131	0.035	0.258	0.809**

Abbreviations: IEM, impressive experience memory; IEM‐W, IEM‐words; SBF, sadomasochist behaviors and fantasies; SBF‐PL, SBF‐play.

** *p* < .01;

* *p* < .05.

It shows that mention of words related to sadomasochism was weakly correlated with that of sex play (*r* = .280, *p* = .047, *p* < .05) and canine play (*r* = .283, *p* = .044, *p* < .05). Mention of words related to sex was moderately correlated with that of sex play (*r* = .496, *p* = .000, *p* < .001) and weakly correlated with that of canine play (*r* = .292, *p* = .037, *p* < .05). Moreover, the mention of words related to canine was weakly correlated with that of canine play (*r* = .366, *p* = .008, *p* < .01). Mention of words related to excretion was strongly correlated with that of sadomasochistic play (*r*= .718, *p* = .000, *p* < 0.001) and more strongly correlated with that of excretion play (*r* = .809, *p* = .000, *p* < .001). Notably, elements related to excrement and excretory organs during the growth stage mentioned by interviewees accurately predicted sadomasochistic behaviors and fantasies and excretion play during adulthood.

##### Correlation between IEM‐P and SBF‐PTN

Correlation analysis was performed on the numbers of RPs of the child nodes of “IEM‐P “and that of “SBF‐PTN,” as shown in Table 16.

**TABLE 16:** Correlation between IEM‐P and SBF‐PTN.

	Personality and temperament	Relationship mode	Appearance	Personal identity and occupation	Age
Parent	−0.089	0.229	−0.057	−0.058	0.263
Other relatives	−0.156	0.214	−0.132	0.070	0.114
Character	0.148	−0.077	−0.033	−0.079	0.064
Teacher	−0.175	0.050	−0.103	−0.025	−0.070
Sex partner	−0.131	0.185	−0.042	0.024	0.114
Childhood playmate	−0.161	0.005	0.227	−0.062	0.210
“Sister” unrelated by blood	0.275	0.057	0.632**	0.863**	0.124
Classmate	−0.124	0.019	−0.142	−0.040	−0.097

Abbreviations: IEM, impressive experience memory; IEM‐P, IEM‐people; SBF, sadomasochist behaviors and fantasies; SBF‐PL, SBF‐play.

** *p* < .01.

It shows that interviewees mentioning “sister” unrelated by blood was strongly correlated with that of partner's appearance (*r* = .632, *p* = .000, *p* < .001) and more strongly correlated with that of partner's identity and occupation (*r* = .863, *p* = .000, *p* < .001). This implies that the more an interviewee mentioned a “sister” unrelated by blood during growth, the more they concentrated on the sadomasochistic partner's appearance and social roles during adulthood.

#### Cluster of “IEM” and “SBF”

**FIGURE 3:**
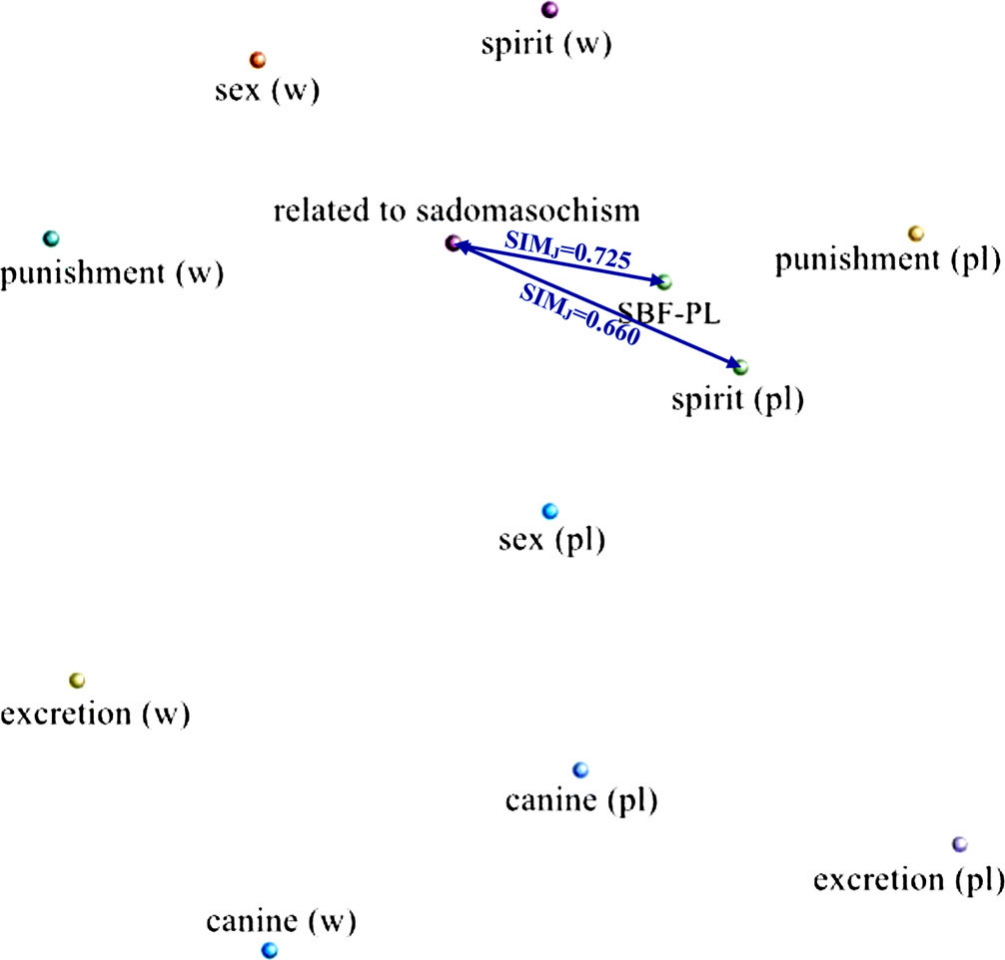
Similarity between “related to sadomasochism” and “sadomasochist behaviors and fantasies (SBF)‐play” and their child nodes.

Cluster analysis by code similarity was performed on nodes at all levels of “IEM” and “SBF.” In the results, only the analysis on the nodes “(words) related to sadomasochism” and “SBF‐PL” and their child nodes showed similarity coefficients greater than 0.6, as shown in Figure 3.

Cluster analysis based on code similarity, at a certain level, reflects the similarity in how the nodes are coded. It can be seen that how the interviewees mentioned sadomasochistic words was highly similar to the way they talked about sadomasochistic play, with a Jaccard similarity coefficient of 0.725, and was highly similar to the way they talked about spirit play, with a Jaccard similarity coefficient of 0.660. This may imply that, for the Chinese interviewees of this study, the way the sadomasochistic elements occurred during growth predicts the way sadomasochistic play, especially spirit play, occurred during adulthood.

### Rational model construction for IEM and SBF based on quantitative and qualitative analysis

**FIGURE 4:**
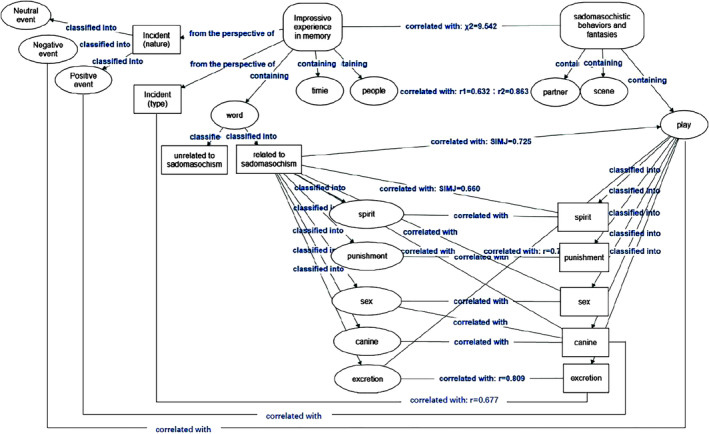
The rational model of impressive experience memory and sadomasochist behaviors and fantasies.

Based on the coding, correlation, and cluster analyses, a rational model of IEM and SBF was constructed using the model construction function of NVivo 12, as shown in Figure 4.

Based on the results of the coding analysis, the relationships between the nodes were preliminarily determined. They were marked as “from the perspective of,” “containing,” “classified into,” and “correlated with” accordingly. IEM could be classified from the perspective of the type and nature of an incident, with the latter classified as positive, neutral, or negative. IEM contains people, time, and word elements, and the word elements could be classified into words unrelated to sadomasochism and that related to sadomasochism, with the latter classified into “spirit,” “punishment,” “sex,” “canine,” and “excretion” words. SBF contains partner, scene, and play elements that could be classified into “spirit,” “punishment,” “sex,” “canine,” and “excretion” play. There were correlations between the people and partner elements, negative events and sadomasochistic play, positive events and canine play, and excretion words and sadomasochistic play. Moreover, there were correlations between the words and play. Statistical values are marked in the model when supported by the Pearson correlation coefficient or Jaccard similarity coefficient greater than 0.6. Correlations with low support will be explored in future studies.

## DISCUSSION

### Conduct of the interviews

#### Interviewees

This was the first time Chinese researchers had conducted interviews on sadomasochism. Limited by the conservative Chinese sex culture, we chose to recruit the participants from the targeted population efficiently on the Chinese sadomasochistic website. We selected interviewees using convenience sampling and recruited 51 interviewees. We did not deliberately recruit interviewees with any quota; therefore, it may not be a representative sample. Our sample failed to transcend the limitations of existing research because no interviewees' sadomasochistic interests violated the safe, sane, and consensual (SSC; Weiss, 2015) principle or met the *DSM‐5* diagnosis criteria according to the interview records, but some interviewees had fantasies of conducting pathological behaviors, such as genital mutilation and “*Binglian* (冰恋),” which is known as generic necrophilia and presented as cutting off meat from a sadomasochistic partner and eating it. Thus, the interviewees in this study could partially represent general sadomasochists. As was stated, studying pathological or non‐pathological sadomasochists brings enlightenment to the other. Still, the representativeness of our sample was limited.

In an Australian national study, there was little difference between the number of men and women, with a ratio of *N*_male_/*N*_female_ = 8628/8185 (Richters et al., 2008). A sadomasochistic sample from the USA had a similar ratio: *N*_male_/*N*_female_ = 801/1033 (Walker & Kuperberg, 2022). Both of these ratios were quite different from the ratio of *N*_male_/*N*_female_ = 49/2 in this study. In some local Chinese social communities, such as the sadomasochistic Tencent Group, there are more men than women. However, the difference was not as apparent as in our sample. Some female users were accustomed to changing their gender data for protection; therefore, the actual Chinese gender ratio may be different. Many women are active in sadomasochistic online communities, but they are less open to participating in research like this.

Most interviewees were aged between 30 and 39 years in this study. Hughes and Hammack's (2020) study included equal numbers of participants in different age ranges, while the youngest participants, aged between 18 and 28 years, formed the largest group in the study by Paarnio et al. (2022). Chinese youth are hardly exposed to sex topics because sex culture is typically conservative, while older sadomasochists might not be familiar enough with information techniques especially when online sadomasochistic communities are so difficult to access. These factors may lead to the lower percentages of interviewees with older and younger ages in our sample. With the remarkable increase of younger netizens in China, the number of young sadomasochists may rise in the future.

Previous studies have rarely compared the number of sadomasochistic roles. A Portuguese study with participants recruited from a sadomasochistic community reported the ratio of *N*_dominant_/*N*_submissive_ = 6/6 (Pascoal et al., 2015). The ratio in a similar sample from Belgium was *N*_dominant_/*N*_submissive_/*N*_switch_ = 61/115/52 (Schuerwegen et al., 2020). The present study identified the interviewees' roles according to their self‐identification. We found similar numbers of sadists and masochists, but there was only one switch. However, according to the interview records, some interviewees' self‐identification might be inconsistent with their sadomasochistic behaviors. For example, one interviewee believed that he was a sadist but had also experienced being tied up by a female sadist and gained sexual satisfaction. In addition, a more significant percentage of switches may exist because some interviewees who self‐identified as sadists or masochists might have had opposite interests, but they might not have tried it, and no such opportunities might have emerged yet. As was noted by Niebudek and Iniewicz (2023), sadomasochists might consider their role to be something acquired through sexual experiences, so we could only use the interviewees' self‐identified roles as a reference.

Previous studies have reported mixed results regarding sadomasochists' education, probably because of the different samples. In this study, interviewees with an education level of or above the undergraduate level were prevalent, similar to Pascoal et al.'s (2015) study, which had an education level ratio of *N*_junior high school_/*N*_high school_/*N*_college_ = 2/11/55, and Coppens et al.'s (2019) study, which had a ratio of *N*_not graduated_/*N*_high school graduate_/*N*_postgraduate_ = 14/64/134. There were 12 interviewees with master's degrees and six with doctoral degrees in this study. However, this does not mean that there was the same education level among the local sadomasochistic group, because sadomasochists with lower education might have had fewer opportunities to see this study's information online.

Only two interviewees (3.922%) had regular sadomasochistic partners. Interviewees with a regular partner were slightly fewer than those without one, in contrast to the findings of the Australian national study (Richters et al., 2008), suggesting that most Chinese sadomasochists do not have stable relationships. However, some interviewees with romantic relationships possibly chose to hide their actual situation to avoid moral pressure. This is because, in Chinese culture, a person with a spouse or partner is not supposed to have another person as their sadomasochistic partner.

#### Interview process

Based on this study's aims and previous studies, and with semi‐structured interviews designed in advance, the scope of the interviews was limited. However, we used open‐ended questions that the interviewees could answer freely. Compared to bottom‐up interviews, our interviews helped obtain organized information and compare it according to existing foreign research. But still, this prevented us from conducting research beyond our framework and obtaining valuable information that we may have overlooked.

This study adopted anonymous online interviews and explained the interview ethics to the website users who were interested in participating. Several users contacted us and said they were “curious about what you want” but “didn't want to be studied” because they thought they deserved a quiet space because of having been treated harshly by the secular world and they thought studying sadomasochists was disrespectful to them. Notably, this study failed to conduct some return interviews to improve coding accuracy because some interviewees cut off their contact with the researchers, though they did not retract consent. Fortunately, other interviewees were still willing to share their thoughts.

It should also be noted that some interviewees might have answered the questions pleasingly and presented answers that they thought the interviewer liked, similar to empathy in psychological counseling. For example, when asked about their partners, some interviewees who self‐identified as masochists answered “professionals like you,” but they might have been fond of other kinds of partners. “Empathy” seems unavoidable because “authority,” usually related to a researcher, a teacher, or similar kinds of people, is a sadomasochistic word. Therefore, interviewees' responses were likely affected.

### Coding of interview records

The total number of words in the interview records was 32,000, which was small compared to general qualitative studies. This is partially because sexual interest is a personal issue, so interviewees were unlikely to provide detailed information. Meanwhile, in complicated situations, abbreviations are often used for convenience. For example, “OTK” is short for “on the knee,” depicting a masochist bending over on a sadist's thigh or knee, being spanked for punishment. “Star” is short for a bondage play called “*star shibaru*,” where a rope is formed to be a star shape on the masochist's chest.

In the secondary coding stage, we coded the records not only by pieces of description, as in existing studies on related topics (Hughes & Hammack, 2020; Walker & Kuperberg, 2022), but also by elements, especially word elements, to form node trees on another level. As we have not observed this process in previous studies, its contribution to this study is debatable.

Tertiary coding has encoded words of IEM and play of SBF in an top‐down manner, which may be limited because it refers to existing frameworks. However, this benefits the categorization of nodes. This study selected the framework of Chinese local sadomasochists' categorization of “spirit,” “punishment,” “sex,” “canine,” and “excretion” instead of existing categorizations (such as Alison et al.'s, 2001, four themes of sadomasochistic play) because they were not comprehensive or distinctive enough and did not conform to the actual situation of Chinese sadomasochists, to whom their own categorization is more relevant. However, their categorization is not perfect. It was used in this study only for expediency. There is no general worldwide classification yet.

### Impressive experience in memory

The most typical impressive memories included those related to family upbringing and sex.

First, family upbringing is an essential experience for sadomasochists, as reported by previous studies (e.g., Z. Li, 2014; Hall, 2014; Hughes & Hammack, 2020). Among the experiences of family upbringing mentioned by the interviewees, being expected to be excellent is quite notable, which might be because expectations pressurize the children. Parents' beating and scolding were the second most common events of family upbringing. Denis (2015) suggests that the connection between mastery and sadism is that the impulse of cruelty arises from the instinct of mastery. Satisfaction linked to an object can check the movement of mastery and thus check the traits of sadism. Other researchers also found that sadomasochism relates to an individual's attachment to an object (Grossman, 2015; Lammers & Imhoff, 2015). When expected to be excellent or beaten and scolded by parents, one fails to obtain satisfaction by mastering the object or developing an attachment to the object—one's parents or oneself—and transforms the drive into a destructive force, that is, sadism or masochism. C. Chen et al. (2015) conducted a questionnaire survey on college students and reported that childhood abuse can positively and directly predict individual aggression. However, in this study, only one interviewee who had been beaten and scolded by his parents identified himself as a sadist, while the other six with the same experience identified themselves as masochists. Abrams et al. (2022) conducted an online questionnaire survey on the regular population via MTurk and reported that childhood abused males were more sexually aroused by the described sadistic behaviors and females by the described masochistic behaviors. But among the five males mentioning parents' beating and scolding during childhood in our sample, only one became a sadist later in adulthood. This implies childhood abuse has various influences on an individual, which decides neither the individual's sadomasochistic role nor the individual's actual sadomasochistic behavior. Or, the influence is merely on sexual fantasies, while in terms of action, the influence might be moderated by other factors.

Second, sex plays a vital role in the growth experience of sadomasochists. Oliveira Júnior and Abdo (2010) pointed out that sadomasochists tend to have more sexual partners and more and earlier sexual experiences. However, how sexual experience drives sadomasochism has been rarely studied. This study specifies these experiences as one's own sex, witnessing others' sex, fetish, intimacy with mothers, and so forth. Notably, intimacy with mothers was mentioned by three male interviewees, while L. Sun (2003) found that a female masochistic poet had an unpleasant relationship with her mother, implying that a male sadomasochist may have experienced intimacy with his mother during growth, while a female sadomasochist may have experienced a bad relationship with her mother.

Studies reporting sadomasochists’ experience related to pornography and other media are quite new (e.g., Walker & Kuperberg, 2022), probably because early studies on sadomasochists' growth history were neither open‐ended investigation nor conducted during the new media age of recent years in which information is conveyed so quickly and conveniently. The experience of watching literary works or websites found in this study along with the nodes of sexual experience, especially “own sex,” “other's sex,” “sex games,” and “notice female image,” indicates that sexual enlightenment is an important part of the interviewees' description. Incidents related to these issues usually happen accidentally. Although no previous studies have focused on sexual enlightenment, this study suggests that operant conditioning and reinforcement occur during the experience of sex or while watching literary works and websites. Therefore, connectionism should be considered in the etiological theory of sadomasochism. According to the behaviorism theory, a connection between sexual satisfaction and sadomasochistic stimulation established during sexual enlightenment and sexual development may trigger sadomasochistic interests.

No interviewees mentioned experiences of animal abuse, being in prison, sexual abuse, bereavement, or social disorder as reported before (Liu et al., 1997; Nordling et al., 2000; Richters et al., 2008; Stupperich & Strack, 2016; Yost & Hunter,2012; Zhao, 2014). This is probably because these experiences are not significant factors of sadomasochists' growth history, though they were mentioned by some case reports or investigations with small samples. Still, there might be an indecent assault from a parent in this study, which was mentioned by one male interviewee who experienced being held very tightly between his mother's legs in early childhood. He thought that he was held asexually at that time, but dimly realized that she did it sexually after growing up.

Similar to what was found in Ten Brink et al.'s (2020) study, no difference was found between the numbers of positive and negative events during interviewees' growth, showing little support for previous studies suggesting that sadomasochists are more likely to experience negative events during childhood (Chen et al., 2015; Hall, 2014; Y. Li et al., 2019; Liu et al., 1997; Nitschke, Osterheider, & Mokros, 2009; Nitschke, Ottermann, & Mokro, 2009; Richters et al., 2008; L. Sun, 2003; Zhao, 2014). Neither negative nor positive experience relates to sadomasochism for sure, but they may affect how sadomasochists perform their sexual interests and the outcome.

Regarding elements extracted from the impressive experience in memory, parents and other relatives were typical character elements, infancy and childhood were typical time elements, and “spirit” words were typical word elements. Obviously, family and childhood play an essential role in the experiences of sadomasochists, implying that psychoanalytic theory is the leading theory for the driving processes of sadomasochism. According to the interview records, although some interviewees tended to attribute their sadomasochistic interests to literary works, sadomasochistic partners, or their own exploration of sex during adulthood, they also emphasized their experiences during infancy and childhood. Family and childhood apparently play important roles in anyone's experience. Still, as was indicated in a previous study on sadomasochists' age when first becoming aware of their inclination (Coppens et al., 2019), it is notable that seeds of sadomasochism are more likely to be planted during one's growth instead of developing from “need satisfaction” or “sensation seeking” in adulthood as some male interviewees believed. This finding shows little support for the hypothesis that sadomasochistic interest simply aims at “entertainment” or “leisure” for pleasure (Williams et al., 2016). Sadomasochists' self‐reported origins of sadomasochistic interests are important clues to understanding how they developed this sexual preference. This has been investigated by existing studies (Hughes & Hammack, 2020; Walker & Kuperberg, 2022), which categorized origins of kink desires based on kinky people's responses to several broad discourses: identity (e.g., personality, personal taste, and role exploration), nurture (e.g., both traumatic and non‐traumatic life experiences), nature (e.g., biology and genetics), culture (pornography, other media and the Internet), negation (e.g., disavowing or doubting a particular idea about the origins of their kink interests), and uncertainty (e.g., not being able to identify an origin of kinky desires). We went through this process, too, during our interviews, and apart from similar results as the above broad discourses, we also found discourse of “curiosity” and “pressure coping.” However, these could only partially provide the basis for clarifying the underlying driving processes of sadomasochistic interests because sadomasochists might be unconscious of the real origin of their sexual interests. Let us say why they did not take other sexual interests as personal taste and why non‐sadomasochists feel disgusting when they accidentally watch sadomasochistic pictures and will not deliberately watch them again. That is why this study tried to test the existing etiological theories of sadomasochism by analyzing the correlation and similarity between growth experience and sadomasochistic behaviors and fantasies rather than based totally on the interviewees' views.

In addition, the present study indicates that inner emotions were an important part of the interviewees' experience, beyond sexual feelings, physical pain, and visual and olfactory feelings. This is quite similar to the inference that psychological abuse plays more roles in the development of sadomasochistic interests from Abrams et al.'s (2022) finding. Finally, “excretion” words are worth noting, given that previous research has neglected this, which is probably because incidents related to excretion are typical experiences of only Chinese sadomasochists.

To collect information on impressive experience in memory, the original question was “What are the most impressive events before adulthood, especially during childhood, in your memory?” After three interviews, the question was changed to “What events are particularly impressive before adulthood, especially in childhood, and especially related to sex, helplessness, and sadomasochistic interests, in your memory?” We revised this based on previous studies to gather more specific information. If the interviewee provided little information for the present question, the interviewer would ask whether there were impressive events unrelated to these themes. However, when asked the theme‐limited question, some interviewees mentioned all the events they could remember regardless of whether they were related to the themes. Some interviewees could not provide much information and remained saying “not any” though they were asked to remember events unrelated to those themes later. Some interviewees answered the question with impressive experiences during adulthood, despite the limited theme of “before adulthood.” This part of the interview was also obtained during the coding process.

Previous studies providing an overview of sadomasochists' growth experiences were mainly case studies (e.g., Liu et al., 1997; Lu, 2004). This study is beyond the scope of a case study and presents more detailed growth experience of sadomasochists than presented before.

### Sadomasochistic behaviors and fantasies

Lack of information about sadomasochistic behaviors and fantasies drives therapists to use their clients to explain sadomasochism for them, which was found to be negative experiences reported by sadomasochists with experiences of psychotherapy (Lantto & Lundberg, 2021). The types of sadomasochistic play mentioned by the interviewees were classified into five categories, known as “spirit,” “punishment,” “sex,” “canine,” and “excretion” play. Previous studies have noted these five kinds of sadomasochistic play (Liu et al., 1997; Pascoal et al., 2015; Renaud & Byers, 1999; Richters et al., 2008; Wignall & McCormack, 2015). Existing studies have not mentioned play other than in these five categories. This categorization differed from the existing ones, such as Alison et al.'s (2001) study or the term “BDSM” containing bondage and discipline, dominance and submission, and sadism and masochism. Existing categorizations are less relevant because it is difficult to specify which category a type of play should belong to. For example, a play in which a sadist collars a masochist and takes him/her for a walk may belong to physical restriction or humiliation according to Alison et al.'s categorization. It may belong to discipline or dominance according to “BDSM.” However, it clearly belongs to “canine” play according to Chinese sadomasochists' categorization. When using existing categorizations, we should consider the purpose and function of the play. It is feasible for Chinese sadomasochists to categorize play into “spirit,” “punishment,” “sex,” “canine,” or “excretion,” which are mutually exclusive, without considering the purpose and function of the play. However, both the general population and foreigners are unfamiliar with this categorization. Thus, they are not easy to apply or popularize. At the beginning of coding, we tried to use the existing categorization, but we failed because the interviews were not detailed enough to classify the words and types of play according to their purpose or function. So, we turned to the Chinese sadomasochist categorization expediently, but a more universal and specific categorization should be developed to promote the comprehension of sadomasochism and to establish rating scales for clinical work.

It is shown that the interviewees liked the “spirit” play the most, emphasizing the structure of the power relationship. Acting dominance and submission is the establishment and exchange of power to perform a dom–sub relationship by changing one's gender or role play. This may support the conclusions of Cross and Matheson (2006), Lammers and Imhoff (2015), and Faccio et al. (2020) that the power relationship between the two sides of the roles is the core of sadomasochism, rather than sex and body abuse, as the public think. Second, it is shown that the “punishment” types of play were the second preference of the interviewees, and that “excretion” play was the least preferred. “Spirit” play is extraordinary and different from “punishment” or “sex” play. When considering the SSC principle (Weiss, 2015), a masochist can hide from or call out the safe word aloud to stop the “punishment” or “sex” play by a sadist. However, a masochist, or more specifically, a submissive, could not do so to hide from or call out the safe word to stop “spirit” play, such as a verbal humiliation from a sadist or a dominant, meaning “spirit” play may be unsafe, insane, and without consent, implicitly.

There was no difference between interviewees' preferences for traditional and oral and anal sex, which differs from Richters et al.'s (2008) finding that sadomasochists were more willing to engage in oral and anal sex than in traditional sex. Traditional sex still occupied many of the interviewees' sadomasochistic behaviors and fantasies. For example, some interviewees integrated traditional sex and “punishment” play together; and some had traditional sex with a fantasy of being scolded. In addition, only two interviewees mentioned the home scene in this study, with only two RPs, whereas Pascoal et al. (2015) found that most sadomasochists implemented their sexual interests at home. Culturally, the interviewees were reserved when implementing this “deviant” sexual interest.

Studying sadomasochistic behaviors and fantasies benefits the evaluation of sadomasochists' mental health and understanding of sadomasochism's function in it. The sadomasochistic phenomenon does not have to be pathological, and it has some kinds of psychological, social, and cultural significance. For example, previous studies have argued that sadomasochism appears to be protective against suicidal ideation (S. Brown et al., 2022); it helps some individuals enjoy leisure (Schuerwegen et al., 2020); it can trigger personal growth, self‐actualization, healing, and transformation (Sprott, 2020); it can undermine patriarchy (Bell, 2013); and it can provide atheists with transcendent experiences (Fennel, 2018). All of this implies that sadomasochism is not always negative. A study even defined BDSM, both in clinical and in community practice, as therapy for mood, stress, or depression (Andrieu et al., 2018). Previous studies have noted that sadomasochism does not require any intervention (Sprott et al., 2017), and it is essential not to pathologize sadomasochists as a starting point of familiarization with their inner world (Iniewicz & Niebudek, 2023). When deciding whether and how to provide professional intervention to sadomasochistic clients, a therapist with adequate knowledge and information about sadomasochistic behaviors and fantasies can better judge whether the client's behaviors and fantasies conform to the SSC principles or reach the *DSM‐5* and other diagnostic criteria. Some types of sadomasochistic play are never conformable to the SSC principle, such as “*Binglian*” (generic necrophilia) and asphyxiophilia. The former occurs more in artistic scenes such as films and novels, while the latter has been reported by case studies in both China and Western countries (e.g., Bauer et al., 2020; Sun et al., 1995). These kinds of play are inconsistent with safety and sane principles, even if both partners consent to it. On the contrary, pathological sadists, known as everyday sadists in previous research, seem to require a non‐consenting target but researchers have found that they endorse sadistic actions regardless of the state of consent (Erickson & Sagarin, 2021), indicating that they could be satisfied by non‐pathological sadism and that their sadistic actions are not always dangerous. This should cause therapists contention, and they must understand what could happen during the various types of play by various sadomasochists. This study has comprehensively collected information about sadomasochist behavior and fantasies; however, it is still incomplete. The minor interest in cutting the partner's flesh for meals and castration can be seen in the interview records. However, some popular types of play performed by Chinese sadomasochists have not appeared in the record, such as speaking or forcing the partner to speak an obscene language, which belongs to “spirit” play.

### The correlation between SBF and IEM

Regarding the correlation between incidents during growth and sadomasochistic play during adulthood, several pairs of node RPs had significant positive correlations. However, most of the correlations were moderate or weak. Only five pairs showed strong positive correlations. Correlation analysis provided quantitative support for the association between interviewees' impressive experience memories and their sexual interests, reflecting the quantitative covariation of the two factors. As it is based on the number of RPs from the coding, this study also performed a cluster analysis to provide qualitative support, which reflected the similarity between the two factors. This study found that two pairs of RPs had strong similarities.

According to the results, a correlation may exist between interviewees' impressive experience memories and sadomasochistic behaviors and fantasies. Notably, in addition to the psychoanalysis and behaviorism theories mentioned above, the concept of “objectives to be fulfilled” in Gestalt theory explains the strong correlation and similarity found in this study. Objectives to be fulfilled will remain until the individual confronts them and resolves them satisfactorily because they were supposed to be fulfilled. Only by reproducing the incidents from the past and experiencing and expressing those negative emotions can they be “fulfilled” (Corey, 2010). The similarity and correlation between impressive experience memories during growth and sadomasochistic behavior and fantasies during adulthood reflect the reproduction of elements of past impressive incidents. They imply that the interviewees tended to fulfill the “objectives to be fulfilled” by performing sadomasochistic interests as another way of expressing their complaint about their parents' upbringing, their desire for self‐mastery and parents' comfort, their hunger for the opposite sex, and so forth. An autoethnographic investigation on BDSM as trauma play has shared that a masochist's trauma play is the practice of intentionally engaging in BDSM activities in order to “play” with his past trauma or abuse and suggested systematic research into this topic (Thomas, 2019). Another study informed by queer‐of‐color critique, feminist performance studies, and psychoanalysis also suggested the reenacting through BDSM of a lesbian/queer individual's own lived trauma in order to reconfigure it (Hammers, 2019). The correlation and similarity emerging from the qualitative data collected from our interviewees kindly support these arguments and initially suggest Gestalt theory for explanation, but more in‐depth data collection is required to explore these hypotheses.

This study combined qualitative and quantitative analyses to explore the correlation between sadomasochism and growth history. However, owing to the limited number of interviewees, there may have been occasional significant strong correlations. For further information about the correlations, we analyzed the interviewees by sex, age, role, education, and presence of a regular partner. This further supports the correlations and indicates that these variables are moderating. We sincerely hope to present our findings in the future. However, to make an accurate conclusion about the correlation, further studies should recruit more samples to collect larger‐scale information.

## CONCLUSION AND IMPLICATIONS

### Conclusion

This study can be summarized as follows:

(1) The most typical impressive experiences in the memories of the interviewees were related to family upbringing and sexual experiences, parents, infancy and childhood period, and “spirit” elements.

(2) The most typical sadomasochistic behaviors and fantasies were related to “spirit” play, the partner's personality and temperament and the relationship mode with the partner, and the outdoor environment.

(3) Significantly more interviewees believed that their sadomasochistic interest was related to their impressive experience in memory. This was supported by the correlation between parents' beating and scolding and excretion play, words related to excretion and sadomasochistic play, words related to excretion and excretion play, “sister” unrelated by blood and sadomasochistic partner's appearance, and “sister” unrelated by blood and sadomasochistic partner's personal identity and occupation, on one hand, and similarity between words related to sadomasochism and sadomasochistic play, and words related to sadomasochism and sprit play, on the other hand.

(4) Psychoanalytic theory is the leading theory for the driving processes of sadomasochism, while behaviorism and Gestalt's theories also contribute to the etiological model of sadomasochism.

(5) A rational model of impressive experiences in memory, sadomasochistic behaviors and fantasies was constructed, showing the relationships among the objectives.

These conclusions call for attention from both parents and professionals.

### Implications

#### Theoretical implications

The rational model of IEM and SBF constructed in this study provides evidence for building a pathological mechanism of sadomasochism consisting of etiology and symptomatology factors. Concerning the etiological factors, the analysis of the interviewees' impressive experience in memory and its correlation with their sadomasochistic behaviors and fantasies indicate the development of sadomasochistic interests be explained based on mastery and attachment from psychoanalytic theory, connectionism from behaviorist theory, and objectives to be fulfilled from Gestalt theory, which has been reflected by some interviewees' views. It kindly implies lacking mastery and attachment builds the foundation of the sadomasochistic trend, while connection and fulfillment impact the ultimate performance of the sadomasochistic trend. Concerning the symptomatological factors, the analysis of the interviewees' sadomasochistic behaviors and fantasies indicates sadomasochistic preference is presented as preferred types of play, partners, and scenes.

#### Practical implications

The rational model of IEM and SBF should be comprehensively considered concerning treatment and healthcare programs for sadomasochists. To explain how sadomasochists develop their sexual interests and provide corresponding professional help, therapists should carefully avoid automatically attributing sadomasochistic interests to the causes they have imagined and collect more information to conclude what growth experience is related to the client's sexual interests. Based on the conclusion of how sadomasochistic interests developed, therapists may consider providing treatment or healthcare according to psychoanalytic, behaviorist, and Gestalt theories or their combination.

Therapists are also supposed to evaluate the risk of being hurt physically and mentally according to the client's preferred types of play. Possible types of play and the risks have been discussed above. For instance, helping a submissive to pick a safe word with the dominant partner is as necessary as helping a masochist with the sadist because a submissive is risking being hurt mentally by spirit play. In addition, like interviewees’ empathy occurring during interviews, clients' empathy could occur during therapy. According to this study, sadomasochistic clients' empathy might be due to their desire to bond with the therapists. Therapists should pay attention to this issue and make good use of it to establish a “working through process,” and avoid barriers during therapy caused by countertransference (Corey, 2010).

According to previous studies and our recruitment process, discrimination against sadomasochists is a widespread phenomenon, which negatively influences research based on sadomasochistic samples and the effect of treatment and healthcare for sadomasochists. This is a complicated ethical paradox in China, mainly because of the large number of child and adolescent netizens and the backward sex education for them, which brings barriers to enhancing the popularization of science on sadomasochistic topics. Therefore, a complete and practical sex education system for Chinese youth is essential. Besides, healthy parenting is the basis of healthy attachment and sex initiation. Chinese parents are lacking parenting guidance for supporting parenting style and a democratic family atmosphere without scolding, especially in rural areas.

## LIMITATIONS AND FUTURE DIRECTIONS

### Limitations

This study has the following limitations:

(1) The sample was formed only by those who were active in online sadomasochistic communities, while those who did not realize their sadomasochistic interests, were not active online, and were sentenced for illegal sadistic behavior or hospitalized for clinically pathological sadomasochism were excluded.

(2) Only two women participated in the interviews, only three interviewees were aged over 40 years, and only four had less than an undergraduate level of education, which resulted in an imperfect sample representativeness.

(3) Sadomasochism has been treated conservatively by the Chinese culture, which might have affected the explicitness of the information collected.

(4) The pre‐designed interview outline might have excluded potentially valuable information.

(5) All analyses were based on the interviewees' descriptions instead of the actual incidents, indicating that it was the typicalness of the content that was studied, which does not always reflect the actual occurrences.

(6) The interviewees' empathy occurring during the interview might have had an impact on the authenticity and accuracy of the data collected.

(7) The categorization of “spirit,” “punishment,” “sex,” “canine,” and “excretion” may be challenging for foreigners to understand.

(8) The interviewees could freely describe sadomasochists' impressive experiences in memory, therefore, the information of growth history collected was not systematic and complete.

(9) Sadomasochistic behaviors and fantasies were analyzed together, so this study failed to understand the difference between behaviors and fantasies and evaluate the ratio of dangerous fantasies changing into actual behavior.

### Future directions

The limited representativeness of our sample suggests that future studies should select samples from patients of psychotic departments and inmates of prisons to analyze the whole group and compare the subgroups. For an accurate ratio of different genders, roles, ages, education levels, and marriage situations, future studies should also collect these data based on a larger sample. Combining samples from online and offline communities and asking about marriage situations through an anonymous questionnaire would be potential options.

An unstructured design of data collection would help detect new information beyond previous research frameworks and perfect the sadomasochistic theory system. Thus, life experience surveys without any limitations are considerable. To collect information without an empathy effect, future studies should adopt methods without contact between the participants and the researchers, such as a questionnaire survey.

We are looking forward to future research that tests the reliability and validity of our design which conducted analysis based on the elements extracted from qualitative material and our categorization of “spirit,” “punishment,” “sex,” “canine,” and “excretion” play. According to our categorization, future studies should also try to develop rating scales or revise diagnosis criteria containing all kinds of play to evaluate implicit, explicit, pathological, and non‐pathological sadomasochism for sadists, masochists, and switches.

Future studies should analyze the growth history by gender and role to find out how gender and other factors moderate growth experience's effect on one's sadomasochistic role and preferred ways to perform sadomasochistic interests. To clarify what roles entertainment and leisure play in sadomasochism, future studies may also deeply explore why the participants choose sadomasochism instead of other types of entertainment. This may further explain the sadomasochistic etiological model formed by the three theories mentioned above. Also, a comparison of the growth experiences of sadomasochists and those of non‐sadomasochists and an exploration of how culture shapes sadomasochistic performance would contribute to this issue.

Previous studies have found that early trauma, such as childhood sexual assault, social avoidance, and sexual disorders, may drive the conversion of dangerous sexual fantasies into actual criminal behavior (e.g., Maniglio, 2010). A reasonable method of interviews and a specific coding system are required to collect and analyze sadomasochistic behaviors and fantasies, respectively.

## CONFLICT OF INTEREST STATEMENT

No potential conflict of interest was reported by the authors.

## ETHICS STATEMENT

This study was approved by the Institutional Review Board of the Institute of Psychology, Chinese Academy of Sciences.

## Supporting information


**Data S1.** Supplementary Information.
