# Supplementary material for: The correlation between sadomasochists' experience and their sadomasochistic behaviors and fantasies: A qualitative analysis of interviews
Source: Psych J. 2023 Dec 17;13(2):295–321. doi: 10.1002/pchj.706 (PMC10990812; doi:10.1002/pchj.706)
Supplement: Supplementary file 1 — Data S1. Supplementary Information. [file PCHJ-13-295-s001.doc]

**Supplementary Material 1**

*Interview questions*

- What are the most impressive events before adulthood, especially during childhood, in your memory? (changed into "what events are particularly impressive before adulthood, especially in childhood, and especially related to sex, helplessness, and sadomasochistic interests in your memory", after three interviews)
- What are the most sadomasochistic plays, partners, and scenes you engage in or with?
- What are the most sadomasochistic plays, partners, and scenes you fantasize?
- Which situation did you feel most satisfied with during sadomasochistic plays?
- In your opinion, does your sadomasochistic behaviors and fantasies associate with the impressive experience before adulthood? If so, what’s the association?
